# Engineered Remolding and Application of Bacterial Membrane Vesicles

**DOI:** 10.3389/fmicb.2021.729369

**Published:** 2021-10-08

**Authors:** Li Qiao, Yifan Rao, Keting Zhu, Xiancai Rao, Renjie Zhou

**Affiliations:** ^1^Department of Emergency, Xinqiao Hospital, Army Medical University, Chongqing, China; ^2^Department of Microbiology, College of Basic Medical Sciences, Key Laboratory of Microbial Engineering Under the Educational Committee in Chongqing, Army Medical University, Chongqing, China

**Keywords:** extracellular vesicles, vesicle production, vesicle immunogenicity, vaccine, delivery system, genetic modification

## Abstract

Bacterial membrane vesicles (MVs) are produced by both Gram-positive and Gram-negative bacteria during growth *in vitro* and *in vivo*. MVs are nanoscale vesicular structures with diameters ranging from 20 to 400 nm. MVs incorporate bacterial lipids, proteins, and often nucleic acids, and can effectively stimulate host immune response against bacterial infections. As vaccine candidates and drug delivery systems, MVs possess high biosafety owing to the lack of self-replication ability. However, wild-type bacterial strains have poor MV yield, and MVs from the wild-type strains may be harmful due to the carriage of toxic components, such as lipopolysaccharides, hemolysins, enzymes, etc. In this review, we summarize the genetic modification of vesicle-producing bacteria to reduce MV toxicity, enhance vesicle immunogenicity, and increase vesicle production. The engineered MVs exhibit broad applications in vaccine designs, vaccine delivery vesicles, and drug delivery systems.

## Introduction

Both eukaryotic and prokaryotic cells can produce extracellular membrane vesicles (MVs), which are nanoscale structures secreted by cells during growth and proliferation (Brown et al., 2015; Gill et al., 2019). Gram-negative (G^–^) bacteria can secret MVs directly from their outer membrane, thus called outer membrane vesicles (OMVs) (DeVoe and Gilchrist, 1973). By contrast, a Gram-positive (G^+^) bacterium has only one cellular membrane covered by a thick layer of peptidoglycan, and its ability to produce MVs was not discovered until Lee et al. (2009). Bacteria can release MVs into the extracellular space in all environments, however they are most easily observed in bacterial culture media (Kaparakis-Liaskos and Ferrero, 2015). The typical MVs are nanoscale bilayer lipid membrane structures with diameters of 20–400 nm (Toyofuku et al., 2019). Bacterial MVs can contain proteins (membrane proteins, lipoproteins, and bacterial toxins), lipopolysaccharides (LPS), and nucleic acids (plasmids, chromosome fragments, and RNA) (Lee et al., 2009; Koeppen et al., 2016; Yuan et al., 2018; Li M. et al., 2020). MVs exhibit important functions, including the transfer of DNA and RNA (Fulsundar et al., 2014; Koeppen et al., 2016), transport of virulence factors (Olaya-Abril et al., 2014; Yuan et al., 2018), interception of bacteriophages (Manning and Kuehn, 2011; Tzipilevich et al., 2017), communication among bacterial populations (Li et al., 2016; Toyofuku et al., 2017), and interaction with host cells (Elmi et al., 2012; Mondal et al., 2016). The inherent characteristics of bacterial MVs make them good candidates for a broad range of applications (Pathirana and Kaparakis-Liaskos, 2016). Firstly, as products secreted by bacterial strains, MVs do not have the ability to grow and reproduce. Thus, the usage of MVs will not cause infections (Kaparakis-Liaskos and Ferrero, 2015). Secondly, plenty of bacterial antigens can be displayed on the surface or sealed inside MVs to stimulate innate and adaptive immune responses. Thus, MVs can be used as vaccines (Parikh et al., 2016; Haque et al., 2021; Li et al., 2021). Thirdly, MVs can incorporate exogenous substances and can easily be used as a vaccine or drug carriers (Tian et al., 2021; Yang et al., 2021). However, some challenges in the application of MVs exist, such as potential biotoxicity (Yuan et al., 2018), insufficient immunogenicity (Wang et al., 2020b), and low natural yield (Cao and Liu, 2020). With the development of bioengineering technology and the deepening knowledge on MVs, further improvement in the safety issue, immunogenicity, or production of MVs is possible through the manipulation of the host bacteria. This review focuses on the engineering of MV-producing bacteria to attenuate MV toxicity, improve MV immunogenicity, and increase MV production. The tremendously improved potential of engineered MVs in vaccine development and drug delivery is discussed.

## Engineering Modification of Bacterial Membrane Vesicles

Membrane vesicles produced by the wild-type bacteria contain toxic components, may carry limited immunogens, and have low yield, resulting in safety problems, inefficiency, and high costs during MV production (Liu et al., 2016a; Cao and Liu, 2020; Yang et al., 2020). Genetic modifications have been widely applied to bacteria for the purposes of MV toxicity reduction, immunogenicity enhancement, and production improvement.

### Detoxification of Membrane Vesicles

Both G^+^ and G^–^ pathogenic bacteria produce toxic molecules that play important roles in bacterial infections (Rivera et al., 2010). As the secreted products of bacteria, MVs may incorporate the toxic molecules during MV formation (Table 1). Detoxification of the toxic components in the MVs is the basic requirement for MV application.

**TABLE 1:** Bacterial virulence factors detected in MVs.

**Bacterium**	**strain**		**MV-loaded virulence factors**	**Ref.**
*Acinetobacter baumannii*	DU202 A38 5806	*132 148 138*	AbOmpA, protease, bacterioferritin, Cu/Zn superoxide dismutase, catalase, ferrichrome–iron receptor Omp38, EpsA, Ptk, GroEL, hemagglutinin-like protein, PgaB FilF	Kwon et al., 2009 Li et al., 2015
*Helicobacter pylori*	CCUG17875 J99 NCTC 11637	*126 162 91*	VacA, CagA, BabA, SabA, AlpB, OipA, urease subunits, HtrA HpaA, Napa HpaA, Napa	Olofsson et al., 2010 Mullaney et al., 2009
*Porphyromonas gingivalis*	W50 33277 W83	151 67 70	CTD proteins, HtrA, HagA, TPR domain protein, RgpA, Kgp FimA, FimR	Veith et al., 2014 Mantri et al., 2015
Enterotoxigenic *Escherichia coli*	jf1412		LT, CexE, EtpA	Roy et al., 2011
Enterohemorrhagic *Escherichia coli*	O157 K-12 O104:H4	66 77	Stx2a, CdtV holotoxins, EHEC-Hly, H7 flagellin, Cytolethal distending toxin A/B/C, Shiga toxin 2 subunit A/B ClyA O104 LPS, ShET1, H4 flagellin	Bielaszewska et al., 2017 Wai et al., 2003 Kunsmann et al., 2015
*Salmonella*	14028s χ3545	*-* 192	PagK1, PagK2, PagJ Flagellin proteins (FlgK, FlgL, FlgC)	Yoon et al., 2011 Liu et al., 2017
*Pseudomonas aeruginosa*	PAO1 PAO1	338 757	EstA, OprF, LasA, OprG, IcmP, OprL, flagellar proteins (FlgK, FlgE) AprA, AlpA/D/E	Choi et al., 2011 Koeppen et al., 2019
*Fusobacterium nucleatum*	EAVG_002	98	MORN2 domain protein, YadA-like domain proteins, AidA, Fap2, FadA,	Liu et al., 2019
*Tannerella forsythia*	ATCC 43037	175	SiaHI, NanH, hemagglutinin, kariysin, CTD proteins (TfsA, TfsB, BspA)	Friedrich et al., 2015
*Campylobacter jejuni*	NCTC 11168	134	CDT, flagellar proteins (flagellin A, B and flagellar hook proteins), CjaA, PorA, Omp50, fibronectin-binding proteins (CadF and Cj1279c)	Jang et al., 2014
*Francisella novicida*	U112	292	Fip, FopB, CyoB, Pcp, RplQ, HtpG, MinD, FumA, LpnA, MaeA, FopC, Pnp, FopA, FipB, Lon, MetlQ, CphA, PutA, CapB, CphB, PdpB, IglI/B/C/D, WbtG/H, FTN_1382, FTN_0714,FTN_0340,FTN_0429,FTN_0643,FTN_0109, FTN_0436, FTN_0325, FTN_0545, FTN_0559, FTN_0597, FTN_0643, FTN_0714, FTN_0855, FTN_0869, FTN_0893, FTN_0925, FTN_1199, FTN_1276, FTN_1277	McCaig et al., 2013
*Moraxella catarrhalis*	Mc6 Mc8	13 14	OMPCD, UspA1, OMPE, OlpA/OmpJ, MID	Augustyniak et al., 2018
*Haemophilus parasuis*	Nagasaki D74	78 84	AidA, OmpP1/2/5, cytolethal distending toxin protein B	McCaig et al., 2016
*Yersinia pestis*	CO92 LCR	270	Ail, Caf1, Pla	Eddy et al., 2014
*Vibrio ordalii*	Vo-LM-18 ATCC 33509^T^		Hemolytic enzyme	Echeverría-Bugueño et al., 2020
*Actinobacillus pleuropneumoniae*	MIDG 2331-Δ*nlpI*	15	RTX toxins (ApxIIA, ApxIIIA, ApxIVA), DegQ, OsmY, Tsp, PtrA,	Antenucci et al., 2019
*Bordetella pertussis*	GMT1		ACT	Donato et al., 2012
*Neisseria meningitids*	NZ98/254	41	PorA	Vipond et al., 2006
*Yersinia pseudotuberculosis*	YPIII	303	CNFy, YopD, YopE, YopH, YopN	Monnappa et al., 2018
*Listeria monocytogenes*	10403s MTCC 1143	- 312	LLO, InlA, InlB, PLC-B, ActA autolysin, P60, PLC-A, PrsA, OppA, murA X- prolyl aminopeptidase, SecDF, SecA2, superoxide dismutase, FlaA	Coelho et al., 2019 Karthikeyan et al., 2019
*Streptococcus pneumoniae*	R6	211	Ply	Olaya-Abril et al., 2014
*Staphylococcus aureus*	8325-4 M060 RN4220	- 85 92	Hla Hld, HlgA/B/C, ETA, ETC, LukD Hld, hlgA/hlgB, SPA	Thay et al., 2013 Jeon et al., 2016 Yuan et al., 2018
*Mycobacterium tuberculosis*	H37Rv	287	SodB, HspX, EphG, Lipoproteins (LpqH, LprA, LprG), PPE41, Rv3722c, Rv0831c, Rv2159c, Rv3099c, Rv3717, Rv3169	Lee et al., 2015
*Staphylococcus epidermidis*	PM221 ATCC12228 RP62A	451 395 518	Glutamyl aminopeptidase, ATP-binding protein OpuCA, LytH, HmrA, FmhA LPXTG-motif cell wall anchor SesE, SesG	Siljamäki et al., 2014
*Bacillus anthracis*	34F2	36	ALO, PA, EF, LF	Rivera et al., 2010
*Enterococcus faecium*	DO E155 K59-68 K60-39	445 351 158 589	AtlA, Acm, CapD, CcpA, Esp, Fnm, PilA2, PrpA, PtsD, SagA, Scm	Wagner et al., 2018

### Detoxification of Membrane Vesicles From G^–^ Bacteria

G^–^ bacteria-produced MVs can have lipopolysaccharides (LPS), adhesins, and other virulence factors (Olofsson et al., 2010; Liu et al., 2016a). As the main component of G^–^ bacterial outer membrane, LPS can stimulate a strong inflammatory response in humans through the toll-like receptor 4 (TLR4)–MD2–CD14 pathway (Kong et al., 2012). The direct incorporation of LPS increases the virulence of MVs and limits their application. MV toxicity could be greatly reduced by altering and modifying the structure of LPS, including acylation and phosphorylation of lipid A, synthesis and transport of core oligosaccharides, and polymerization of O-antigen polysaccharides (Yang et al., 2020).

Lipopolysaccharides consists of lipid A, core oligosaccharide, and O-antigen (Raetz and Whitfield, 2002). Lipid A, which is the toxic group of LPS, consists of a hexacylated diglucosamine, six acyl chains, and two phosphate groups (Raetz et al., 2007). LpxM, LpxL, and PagL are vital acyl transferases involved in lipid-A modification in bacteria, such as *Escherichia coli* and *Salmonella* (Raetz et al., 2007; Bertani and Ruiz, 2018). Ranallo et al. (2010) reported that the deletion of *msbB* (*lpxM*) in *Shigella flexneri* results in the formation of penta-acylated lipid A, which could serve as a TLR4-antagonist. The mortality rate was reduced to 37–50% in mice challenged with penta-acylated lipid A for 72 h compared with mice challenged with wild-type lipid A (100%) (Ranallo et al., 2010). Lee et al. (2017) demonstrated that the mice infected with the phosphatase gene *lpxF*-mutated *E. coli* exhibited less weight loss and slighter lung inflammation than the mice infected with the wild-type strain. To eliminate the effect of LPS in the MVs, the *lpxL1* gene for LPS biosynthesis in *Neisseria meningitidis* was genetically deleted, and the toxicity of MVs was attenuated (van de Waterbeemd et al., 2010). However, the growth rate of the *lpxL1* mutant was remarkably affected compared with that of the wild-type strain. Therefore, this mutant may not be suitable for application due to the growth defect.

In most G^–^ bacteria, the genes for core oligosaccharide and O-antigen synthesis are integrated into two operons, namely, *waa* and *wba* (*rfb*) (Whitfield et al., 2003; Frirdich and Whitfield, 2005). The lack of full-length O-antigens and/or incomplete core polysaccharides leads to the truncation of LPS (Liu et al., 2016b). Liu et al. (2016a) found that the mice infected with MVs produced by *waaC*-, *rfaH*-, or *rfbP*-deleted *S. Typhimurium* mutants presented a higher survival rate of 16.7–33.3% compared with the mice inoculated with MVs produced by the wild-type strain. The incomplete structure of LPS caused by the engineered remolding of the key genes for LPS biosynthesis is an important way to attenuate MV toxicity, but such approaches have usually let to decrease in MV yield. Therefore, optimizing the strategy to achieve knock-out mutants with normal growth is a quite significant issue for application of the engineered MVs.

In addition to LPS, MVs from G^–^ bacteria can package numerous other virulence factors, such as bacterial adhesins, proteases, and cytotoxins (Table 1). Li et al. (2021) consecutively deleted 14 genes encoding variant virulence factors in *Pseudomonas aeruginosa* PA103 to generate a PA-m14 mutant (Δ*exoU*/Δ*exoA*/Δ*exoT*/Δ*lasA*/Δ*lasB*/Δ*wbjA*/Δ*pchA*/Δ *phzM*/Δ*alg*/Δ*RhlAB*/Δ*pvdA*/Δ*plcH*/Δ*phoA/*Δ*lpxL*). The sizes of MVs produced by PA-m14 were greatly smaller than those from the wild-type PA103. Intramuscular injection with 50 μg MVs from PA-m14 mutant did not cause any death in BALB/c mice, in contrast 100% of mice challenged with wild-type MVs died after 3 days. Such consecutive deletion of genes encoding different virulence factors in bacteria is an effective strategy to attenuate bacterial MVs, whereas this method takes time and effort. To prepare MVs with reduced toxicity, new fast and effective strategies for bacterial engineering are urgently needed.

### Detoxification of Membrane Vesicles From G^+^ Bacteria

In G^+^ bacteria, the genetic manipulation of genes for virulence factors may result in the detoxification of MVs. In *Staphylococcus aureus*, the expression of virulence factors is controlled by a complex regulatory network that responds to host and environmental changes. The well-studied regulatory elements in *S. aureus* strains are the accessory gene regulatory system (Agr) and the SaeR/S two-component system (SaeR/S TCS). Agr encodes a quorum sensing system to control the expression of major virulence factors, including exotoxin up-regulation and surface protein down-regulation (Jenul and Horswill, 2019). The SaeR/S TCS consists of four genes (*saeP*, *saeQ*, *saeR*, and *saeS*) controlled by two promoters (P1 and P3), which play a major role in regulating the production of more than 20 virulence factors in *S. aureus* (Liu et al., 2016c). In a study conducted to determine the effect of Agr and SaeR/S TCS on the virulence of *S. aureus*-secreted MVs, Yuan et al. (2014) found that the mortality of mice challenged with engineered MVs derived from *S. aureus* strain RN4220-Δ*agr* was remarkably reduced compared with that of mice stimulated with wild-type MVs. In addition, Wang et al. (2018) found that the single mutant of global regulator *agr* in *S. aureus* strain JE2 (JE2Δ*agr*) remarkably reduced the mRNA expressions of genes that encode all nine subunits of staphylococcal leukocidins and the gene *hla* that encodes alpha toxin. Immunization of female Swiss Webster mice with 5μg MVs produced by the double mutant of *agr* and *spa* (encoding protein A) in *S. aureus* JE2 (JE2-Δ*agr*Δ*spa*) provided significant protection against fatal sepsis caused by a heterologous USA300 isolate, FPR3757 (Wang et al., 2018). MVs produced by *S. aureus* JE2-Δ*agr*Δ*sae* remarkably reduced the cytotoxicity to THP-1 macrophages (Wang et al., 2020a). Thus, engineering bacteria by deletion of regulatory systems controlling virulence gene expression is promising for MV detoxification.

### Enhancement of Membrane Vesicle Immunogenicity

**FIGURE 1:**
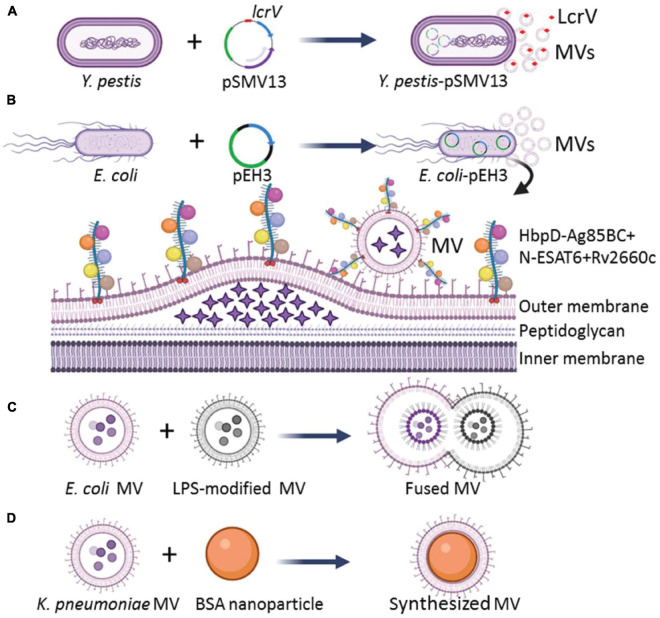
Strategies for engineering of bacterial MVs with enhanced immunogenicity. **(A)** Engineering *Y. pestis* by transformation of an Asd^+^ plasmid pSMV13 to express antigen LcrV, and MVs produced by the engineered *Y. pestis* loaded more LcrV than those derived from the wild-type strain. **(B)** *E. coli* transformed with a recombinant plasmid pEH3 to express HbpD^+^ *M. tuberculosis* Ag85B_C+N_-ESAT6 + Rv2660c chimeric antigens, and the expressed HbpD-Ag85B_C+N_-ESAT6-Rv2660c chimeric antigen could be effectively integrated into the MVs. The asterisks in the lumen of MVs represent bacterial proteins. **(C)** The MVs with enhanced immunogenicity could be prepared *in vitro* by the aggregation and fusion of MVs derived from different bacterial strains or species. The circle dots colored with diverse blue in the lumen of MVs represent bacterial proteins, DNA, and RNA. **(D)** Larger MVs could be constructed by depositing of MVs onto BSA nanoparticles.

The immunogenicity of MV-contained antigens is crucial to the successful development of an MV vaccine (Yuan et al., 2018). The key to inducing an effective immune response is the ingestion of antigen-containing particles by antigen-presenting cells (APCs). Therefore, engineering bacteria to load more target antigens into MVs and manipulating MV nanoparticles are effective ways to enhance MV immunogenicity. Here, we discuss four useful strategies applied to enhance the immunogenicity of MVs, which improved the application prospect of MVs (Figure 1).

First, overexpression of the protective antigens in a harmless bacterium was used to generate MVs with reduced toxicity. The immune response induced by *Yersinia pestis* MVs depends on the immune dominant LcrV. To strengthen the immunogenicity of MVs, Wang et al. (2020b) constructed a host-vector balanced lethal system based on an essential bacterial gene encoding aspartate β-semialdehyde dehydrogenase (Asd) to overexpress the LcrV antigen of *Y. pestis* and to reduce bacterial toxicity. The wild-type *Y. pestis* isolates carry a virulent plasmid pCD1 with genes to encode virulence effectors (YopE, YopJ, YopH, YopM, and YopT) and protective antigen LcrV. The pCD1-deficient *Y. pestis* was engineered to overexpress the LcrV antigen with Asd^+^ plasmid pSMV13 carrying a chimeric gene to encode the N-terminal-lactamase signal peptide fused with LcrV (Figure 1A). Increased amounts of LcrV antigen enclosed in the MVs were observed. The engineered MV-immunized mice produced high titers of IgG against LcrV and higher levels of Th1 cytokines (IFN-γ, IL-2, IL-17, and TNF-α) than those from recombinant LcrV/alhydrogel-immunized mice (Wang et al., 2020b). Therefore, the overexpression of LcrV antigen with a recombinant plasmid in pCD1-free *Y. pestis* can significantly enhance the immunogenicity of MVs.

Antigens of pathogens are fused with the MV-loaded proteins of otherwise harmless bacteria to mitigate toxicity but still induce a specific immune response. Several bacterial proteins, such as *E. coli* ClyA (a hemolytic protein that forms small pores) (Chen et al., 2010), adhesin involved in diffuse adherence (AIDA-I) auto-transporter domain (Rizos et al., 2003), hemoglobin protease (Hbp) (Daleke-Schermerhorn et al., 2014), and *N. meningitidis* factor H binding protein (FHbp) (Shirley and Taha, 2018; Findlow et al., 2020) were studied for their potential to carry and display heterologous antigens on the MV surface. Hbp consists of an N-terminal cleavable signal sequence, a secreted passenger domain, and a C-terminal β-domain. Mature Hbp often folds into ∼100-Å β-helical stem structure, which acts as a stable scaffold for the five salient lateral domains (D1 to D5) (Otto et al., 2005). Daleke-Schermerhorn et al. (2014) replaced the side domains (D1, D2, D4, and D5) with *Mycobacterium tuberculosis* antigens Ag85B_C_, Ag85B_N_, ESAT6, and Rv2660c, respectively, and the fusion protein HbpD-Ag85B_C+N_-ESAT6-Rv2660c was successfully expressed in the recombinant *E. coli*. All *M. tuberculosis* antigens were loaded to the surface of *E. coli* MVs by the Hbp auto-transporter platform (Figure 1B). The engineered MVs could induce a CD4^+^ T cell response against the *M. tuberculosis* infections in mice (Prados-Rosales et al., 2014a). van den Berg van Saparoea et al. (2018) modified the Hbp display platform with a SpyTag/SpyCatcher protein ligation system. The fusion of SpyTag to the Hbp did not impair the display on bacterial MVs, and the addition of purified proteins fused to the SpyCatcher domain could efficiently couple to Hbp-SpyTag. Thus, multiple antigen modules (SpyCatcher domain-fused) could easily be ligated to Hbp on MVs. Such engineered MVs could effectively stimulate the immune response (van den Berg van Saparoea et al., 2018, 2020). Therefore, the fusion of multiple antigens of a pathogen to the MV-contained bacterial transporters and proteins is another effective strategy to increase immunogenicity of the MVs.

The diversity of heterogeneous antigens loaded by bacterial MVs can be enhanced by the fusion of MV populations. Aggregation and fusion can be performed with MVs from different bacterial strains or species. Gnopo et al. (2020) prepared the MVs of native *E. coli* Nissle strain 1917 (EcN MV) and its LPS-modified strain ClearColi (CC MV). Then, the aggregation and fusion of MVs were performed by adding equal volumes of EcN MV and CC MV and inducing at low pH value of 3.6, as well as modulating ion composition and concentration to form a multifunctional vesicle (Figure 1C). The fusion efficiency approached ∼25%, and the MV-fusion strategy facilitates the design of multi-antigen vaccines that can elicit effective immune responses (Gnopo et al., 2020). A high fusion efficiency of bacterial MVs may be achieved with decreased pH and increased salt concentration (Gnopo et al., 2020), however, the optimizing conditions for an ideal fusion efficiency needed to make large-scale applications, as well as the detailed composition and architecture of the fused vesicles require further investigation.

The size, shape, and rigidity of MV nanoparticles can affect the APC uptake, antigen presentation, and activation (Benne et al., 2016). Adjustment of the properties of MV nanoparticles is also a valuable strategy to enhance the immune response. Shima et al. (2013) found that when poly γ-glutamic acid-graft-L-phenylalanine (γ-PGA-Phe) nanoparticles with sizes of 40, 100, and 200 nm were subcutaneously injected into mice, respectively, the 40 nm nanoparticles distributed more rapidly to lymph nodes of the challenged mice and were taken up by a greater number of dendritic cells (DCs) compared with the 100 and 200 nm γ-PGA-Phe nanoparticles. This finding indicates that smaller-sized nanoparticles are taken more effectively by APCs than larger-sized ones. Therefore, the immune effect of MV nanoparticles can be maximized by properly controlling the nanoparticle sizes. Wu et al. (2020) deposited the hollow-structured MVs produced by carbapenem-resistant *Klebsiella pneumoniae* onto 70 nm bovine serum albumin (BSA) nanoparticles (BN) to synthesize 100 nm BN-MV by a mechanical extrusion process (Figure 1D), and the BN-MV increased the expression of CD11c, CD40, CD80, CD86, and MHC-II by cell line of DC 2.4 compared with those stimulated with the wild-type bacterial MVs. Taken together, the structure optimization of MV nanoparticles can effectively improve the immune efficacy of bacterial MVs for vaccine development.

### Improvement of Membrane Vesicle Production

When considering MVs for medical applications, MV yield of bacteria under natural conditions is generally low, which is one of the most important factors limiting MV application (Wang et al., 2018; Cao and Liu, 2020). MV production can be increased by regulating bacterial growth, increasing the accumulation of components in the bacterial outer membrane, changing the fluidity of the cell membrane, and reducing the degree of cross-linking of peptidoglycan (Toyofuku et al., 2019).

There is increasing evidence that MV production is strongly affected by the growth conditions of bacteria. Many environmental factors influence the rate of bacterial MV formation, including media composition, growth phase, culture temperature, iron concentration, oxygen availability, and antibiotics exposure (Kulp and Kuehn, 2010; Orench-Rivera and Kuehn, 2016). For example, a study conducted by Choi et al. (2014) revealed that MV production of *Pseudomonas putida* KT2440 in Luria Bertani (LB) broth was increased more than three-fold than that in the minimal medium with 10 mM succinate or minimal medium with 5 mM benzoate. Anoxic cultures of *P. aeruginosa* PAO1 with LB media produced up to six-fold more MVs in comparison to the aerobic conditions (Toyofuku et al., 2014). In the cases of *Helicobacter pylori* and *M. tuberculosis*, MV productions were enhanced in iron limiting conditions (Keenan and Allardyce, 2000; Prados-Rosales et al., 2014b). Maredia et al. (2012) demonstrated that *P. aeruginosa* treated with ciprofloxacin increased MV production by 100-fold in comparison to the untreated bacteria. When treated with β-lactam antibiotics (flucloxacillin and ceftaroline), *S. aureus* increased the MV production in both a lysogenic and a virus-free strain. Ciprofloxacin triggered MV production in the lysogenic *S. aureus* isolates but not in their phage-free counterparts (Andreoni et al., 2019). Optimizing the conditions to increase bacterial MV production may be strain- or species-dependent, however, it is worth to be investigated for MV yield improvement.

In addition to environmental factors, lots of bacterial molecules were found to be associated with MV production. Genetic manipulation of certain molecules in target bacteria has been performed to greatly improve MV production (Table 2). A study by Obana et al. (2017) revealed that the spore formation pathway of *Clostridium perfringens* is related to MV production. The phosphorylation of a conserved aspartic acid residue (Asp58) in the Spo0A protein encoded by the spore formation regulatory gene *spo0A* is essential for MV production. Meanwhile, sporulation-related sensor kinases promote the MV production. Sensor kinases, such as CPE1316 and *ReeS*, can regulate the production of MVs through the phosphorylation of the *C. perfringens* Spo0A protein. MV production of *spo0A* knock-out strain is reduced by about five times compared with the wild-type strain, while overexpression of the *spo0A* gene in *C. perfringens* increases MV production by four times (Obana et al., 2017). In Group A *Streptococcus* (GAS), the CovRS two-component system negatively regulates the production of MVs. Deletion of the *covRS* gene in GAS increased MV production (Resch et al., 2016). In *M. tuberculosis*, MV production is regulated through a Pst/SenX3-RegX3 signal transduction pathway (White et al., 2018). Knock-out of the *pstA1* gene, which encodes the membrane-spanning component of the phosphate-specific transport (PST) system, weakened the inhibitory effect of the PST system, and resulted in the activation of SenX3-RegX3 two-component system and an approximately 15-fold increase in MV production (White et al., 2018). Wen et al. (2021) used functional genomics to identify genes associated with MV production in *Streptococcus mutans* and found that *sfp*, *bacA*, *bacA2*, *dac*, and *pdeA* genes affected bacterial MV production. In *Listeria monocytogenes*, the MV yield of the *sigB*-mutant strain is approximately nine times lower than that of the wild-type strain (Lee et al., 2013).

**TABLE 2:** Genetic modification of target molecules that affect MV production.

**Bacterial species**	**Genetic modification and/or culture condition**	**Improved yield relative to the wild-type strain or normal condition**	**Methods for MV quantification**	**Ref.**
*Haemophilus influenzae*	Δ*vacJ* Δ*yrbE*	1.6-fold increase 2.2-fold increase	Braford protein assay	Roier et al., 2016
*Vibrio cholerae*	Δ*vacJ* Δ*yrbE*	3.9-fold increase 4.3-fold increase	Braford protein assay	Roier et al., 2016
*Neisseria meningitidis*	Conversion of batch to continuous processes	8.9-fold increase	Lowry protein assay	Gerritzen et al., 2019
*Shigella sonnei*	Δ*tolR* High density culture	Increase 1.8-fold increase	SDS-PAGE Braford protein assay	Berlanda Scorza et al., 2012
*Escherichia coli*	Δ*tolR* Δ*tolA*, ultradiafiltration Δ*tolA*, ultracentrifugation Δ*degS* Δ*degP* Δ*Dlm*	32.9-fold increase 51.9-fold increase 77.8-fold increase 7.8-fold increase 3.5-fold increase 12–19-fold increase 5.6-fold increase 4.8-fold increase	Lowry protein assay Purpald LPS assay FM4-64 assay NTA NTA SDS-PAGE SDS-PAGE SDS-PAGE	Pérez-Cruz et al., 2016 Reimer et al., 2021 McBroom and Kuehn, 2007 Pasqua et al., 2021
*Pseudomonas putida*	a chain length of C7 and longer in *n*-alkanols	2–4-fold increase	Braford protein assay	Eberlein et al., 2019
*Acinetobacter baumannii*	Δ*bfmS* Sucrose-extracted MV ΔAbOmpA	4.5-fold increase 8.8-fold increase 13.2-fold increase 7.3-fold increase	BCA protein assay Lowry protein assay BCA protein assay *Limulus* Amebocyte lysate test	Kim et al., 2019 Li S. et al., 2020 Moon et al., 2012
*Campylobacter jejuni*	Δ*mlaA*	1.7-fold increase 1.5-fold increase	KDO assay BCA protein assay	Davies et al., 2019
*Pseudomonas aeruginosa*	Δ*oprI* Δ*oprF*	3-fold increase 8-fold increase	Phospholipid assay	Wessel et al., 2013
*Serratia marcescens*	Δ*wecD*	5-fold increase	KDO assay	McMahon et al., 2012
*Bacillus subtilis*	*sfp* loss of function Δ*xhlAB*/Δ*xlyA* Δ*lytCDEF* SFE treatment Cold shock Starvation Low O_2_	5.2-fold increase No effect Loss response to stress condition 10-fold increase 13-fold increase 22-fold increase 8-fold increase	^14^C assay FM1-43 assay	Brown et al., 2014 Abe et al., 2021
*Streptococcus mutans*	Δ*sfp*	1.7-fold	BCA protein assay	Wen et al., 2021
*Mycobacterium tuberculosis*	Δ*pstA1* Δ*virR*	15-fold increase 1.5-fold increase	NTA Braford protein assay	White et al., 2018 Rath et al., 2013
*Staphylococcus aureus*	Δ*psm*α1-4 *sle1* overexpression Δ*pbp4* Δ*tagO* Δ*psm*α Δ*lgt* Δ*agr*, linoleic acid treated	3.5-fold decrease Increase 3.0-fold decrease Increase Decrease 2-fold increase 70-110% decrease	BCA protein assay FM4-64 assay BCA protein assay FM4-64 assay	Wang et al., 2018 Schlatterer et al., 2018 Wang et al., 2020a Kengmo Tchoupa and Peschel, 2020
Group A *Streptococcus*	*covRS* loss of function	5.2-fold increase	FM1-43 assay	Resch et al., 2016
*Listeria monocytogenes*	Δ*sigB*	9-fold decrease	BCA protein assay	Lee et al., 2013
*Clostridium perfringens*	Δ*spo0A* Δ*CPE1316* Δ*rees*	5-fold decrease 3-fold decrease 3.5-fold decrease	BCA protein assay	Obana et al., 2017

*KDO, 3-deoxy-D-manno-octulosonic acid; NTA, Nanoparticle tracking analysis; BCA, modified bicinchoninic acid; SFE, sucrose fatty acid ester.*

**FIGURE 2:**
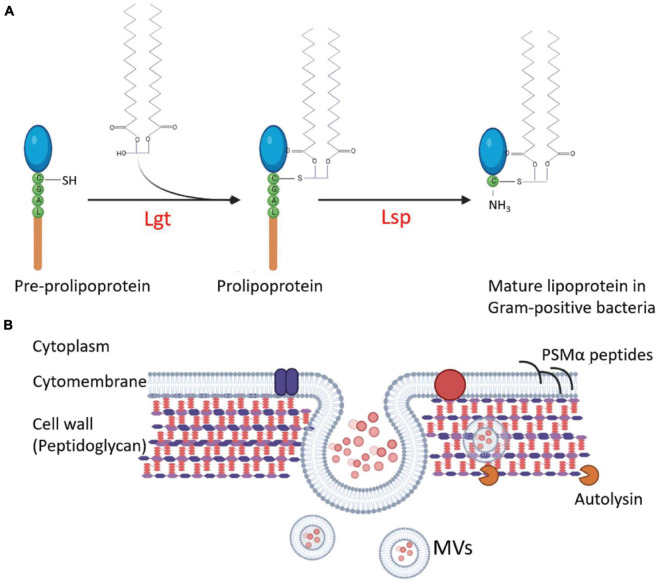
Regulation of the MV production in G^+^ bacteria. **(A)** Interference of lipoprotein maturation increases MV production. During lipoprotein maturation of G^+^ bacteria, the sulfhydryl group of prolipoprotein cysteine is modified and lipidated by lipoprotein diacylglyceryl transferase (Lgt) and then the lipoprotein signal peptidase (Lsp) cleaves the signal peptide to make cysteine a new amino terminal residue to form mature lipoproteins. Knocking out of *lgt* increases MV production of the *S. aureus* mutant. **(B)** The strategies involved in the regulation of MV production in *S. aureus*. The MVs are produced from the plasma membrane of *S. aureus*, and staphylococcal lipoproteins can be mobilized from the cell membrane to increase the fluidity of the membrane and promote MV formation. At the same time, MVs must traverse the highly cross-linked peptidoglycan layer to release. The degradation of the peptidoglycan layer or the reduction of cross-linking promotes MV yield. Autolysins, such as Sle1, promote MV release by hydrolyzing peptidoglycan of *S. aureus* cells. The circle dots colored with diverse pink in the lumen of MVs represent bacterial proteins, DNA, and RNA.

The first step in the release of MVs is the budding of the cell membrane, which can be promoted by altering cell membrane fluidity and lipoproteins, which play important roles in maintaining fluidity. Schlatterer et al. (2018) found that *S. aureus* MVs contain many cytoplasmic proteins, and phenol-soluble modulins (PSMs) can mobilize lipoproteins from the cytoplasmic membrane to increase membrane fluidity, resulting in the formation of MVs. In the *agr*-deficient *S. aureus* strain SA113 that does not express PSMs or *psm*α1-4 gene-deleted strain USA300, the MV yield is substantially decreased. Overexpression of *psm*α1-4 genes in *S. aureus* SA113 with a vector pTX16-*psm*α1-4, the MV release of recombinant strain increased 4.2-fold compared with that of the SA113 carrying an empty pTX16 (Schlatterer et al., 2018). Meanwhile, the lack of lipoproteins can increase cytoplasmic membrane fluidity. Lipoprotein diacylglyceryl transferases (Lgt) catalyze the acylation of lipoproteins and play an important role in lipoprotein lipidation and maturation (Figure 2A; Kovacs-Simon et al., 2011). After the knock-out of *lgt*, the production of *S. aureus* MVs increases (Wang et al., 2020a). Deletion of the *tolR* gene in *E. coli* IHE3034 results in substantial increase of MV production without loss of membrane integrity (Berlanda Scorza et al., 2008).

The highly cross-linked peptidoglycan (PGN) layers are the main barrier for MV release, mostly in G^+^ bacteria. The methods for PGN degradation or reduction of the cell wall cross-linking may promote the production of MVs. After treating *S. aureus* with a sublethal concentration of penicillin G (PenG), the PGN cross-linking decreased, and MV yield increased by about 10 times compared with the untreated strain (Wang et al., 2018; Andreoni et al., 2019). The deletion of genes associated with bacterial cell wall synthesis, such as *pbp4* and *tagO*, which encodes an N-acetyl glucosamine-phosphate transferase enzyme to catalyze the biosynthesis of wall teichoic acid (a PGN-anchored glycopolymer and a major component of the *S. aureus* cell wall), can also lead to a decrease in the cross-linking of *S. aureus* PGN and a 3-fold to 4-fold increase in MV production (Wang et al., 2018). The *sle1* gene product is a PGN hydrolase of *S. aureus* strains (Figure 2B). Deletion of *sle1* in *S. aureus* reduced MV production. When *sle1* was overexpressed, MV production could remarkably be increased (Wang et al., 2018). In addition, many endolysins produced by bacteriophages have PGN hydrolase activities. During bacteriophage biosynthesis in a bacterial cell, the endolysins can destroy the cell wall from the inside to facilitate MV release and to promote MV production (Toyofuku et al., 2017).

During MV production, the stability of MVs is a crucial hurdle for their application. The antigens in bacterial MVs could be released through the destruction of lipid membrane of MVs by surfactants or functional enzymes. Reducing MV damage may promote their accumulation and result in a high yield. Sfp is a 4′-phosphopantetheinyl transferase; it is crucial in lipopeptide surfactant biosynthesis in *Bacillus subtilis*. Brown et al. (2014) found that the MV yield of *B. subtilis* strain harboring a functional *sfp* gene was less than that of a strain with non-functional *sfp* gene. PSMs can promote MV production in *S. aureus* (Wang et al., 2018). However, PSMs have surfactant-like activities, they can destroy MVs at the concentrations more than 12.5 μg/ml (Schlatterer et al., 2018). This finding correlates with the fact that *S. aureus* MVs are mostly isolated from culture supernatants collected between 6 and 8 h of cultivation when the PSM concentration in cultures was below 12.5 μg/ml (Schlatterer et al., 2018). Therefore, avoidance of disruption is another important issue in the large-scale preparation of MVs for application.

## Application of the Engineered Bacterial Membrane Vesicles

Although the detailed mechanisms underlying the formation of bacterial MVs are still not fully elucidated (Chen Q. et al., 2020), the application of either naturally produced or engineered bacterial MVs is promising mostly in vaccine development and delivery system construction.

### Vaccine Development

Vaccines are suspensions of inactivated, weakened, or fragmented toxic antigens or disease-causing agents, such as bacteria, viruses, and parasites or of antibodies or lymphocytes that are vaccinated for disease prevention. In the field of bacterial MV vaccines, *N. meningitidis* MV vaccine is the most widely studied. Two meningococcal serogroup B (MenB) MV vaccines, namely, MenB-4C and MenB-FHbp, have been currently approved in Europe to prevent invasive meningococcal disease (IMD) (Basta et al., 2016; Parikh et al., 2016; De Wals et al., 2017; Grogan and Roos, 2017; Rappuoli et al., 2018; Shirley and Taha, 2018). The response induced by the monovalent MV vaccine is ascribed to the immune dominant PorA, which has a high degree of sequence diversity and has limitations in covering various MenB strains. FHbp is a surface-exposed protein that is widely distributed in the meningococcal isolates and has high immunogenicity. The MenB-4C and MenB-FHbp MV vaccines could protect against infections caused by the 14 pathogenic meningococcal strains tested (Findlow et al., 2020). Li et al. (2021) prepared *P. aeruginosa* MV vaccine (OMV-PH) enclosed the recombinant PcrV-HitAT (PH) bivalent antigen. Vaccination with this engineered OMV-PH vaccine in BALB/c mice exhibited 70% protection from the intranasal infection with 6.5 × 10^6^ colony forming unit of *P. aeruginosa* PA103, while immunization of mice with MVs in absence of PH antigen failed to afford effective protection against the same dose of PA103 challenge (Li et al., 2021).

The MVs from *Streptococcus pneumoniae* and *M. tuberculosis* are rich in bacterial lipoproteins, which can induce humoral immunity to produce antibodies against infections caused by *S. pneumoniae* and *M. tuberculosis*, respectively (Olaya-Abril et al., 2014; Prados-Rosales et al., 2014a). Vaccination with *S. aureus* MVs can activate Th1 and Th17 cells to induce cellular response in mice and can also stimulate B cells to produce antibody response against *S. aureus* infection (Choi et al., 2015). In addition, staphylococcal MVs can up-regulate the expression of co-stimulatory molecules, such as IL-12 and IL-6. Wang et al. (2018) prepared highly immunogenic attenuated MVs by knocking out the genes *agr* and *spa* and expressing non-toxic HlaH35L and LukE antigens in the engineered *S. aureus* strain. Such engineered MVs elicited effective protection against lethal sepsis caused by *S. aureus* strain USA300 LAC (Wang et al., 2018). Both naturally occurring bacterial MVs and engineered MVs can be developed as new vaccines.

### Vaccine Delivery Vehicle

The powerful delivery capabilities of MVs for exogenous antigens make bacterial MVs promising vaccine delivery vehicles. The MVs produced by G^–^ bacteria for loading native antigens, heterologously expressed proteins, or fused molecules on the surface and in the lumen have been extensively investigated. Kesty and Kuehn (2004) tested whether a heterologously expressed protein would be delivered into *E. coli* MVs. They expressed an outer membrane adhesin Ail from *Yersinia enterocolitica* in *E. coli* strains DH5α, HB101, and MC4100 with a recombinant plasmid encoded Ail. The Ail was successfully delivered into the MVs of all three strains tested. The authors proposed that the expressed exogenous proteins were firstly secreted into the periplasmic space of bacteria. MVs might take in the heterologous proteins from the periplasmic space and integrate them into the vesicles during MV release and maturation. To evaluate the delivery efficiency of exogenous antigens, several MV-enriched endogenous molecules were screened as carriers to deliver vaccine candidates by a protein fusion strategy. *E. coli* ClyA, Hbp, AIDA, *N. meningitidis* FHbp, and *S. aureus* Mntc, Eno, and PdhB are experimentally verified bacterial molecules capable of delivery of foreign antigens (Benz and Schmidt, 1989; Chen et al., 2010; Daleke-Schermerhorn et al., 2014; Yuan et al., 2018). Huang et al. (2016) constructed a ClyA-Omp22 fusion protein in *E. coli* strain DH5α, and the MVs produced by the engineered bacteria contained the Omp22 antigen of *Acinetobacter baumannii*. The mice immunized with the engineered MVs produced a strong Omp22-specific humoral immune response that protect mice from lethal *A. baumannii* attacks (Huang et al., 2016). Yang et al. (2021) fused the receptor binding domain (RBD) of SARS-Cov-2 to ClyA and expressed the Cly RBD protein in *E. coli* BL21, then a bacterial biomimetic vesicle (BBV) was generated with MVs of the engineered bacteria extra loaded with polymerized RBD (RBD-BBV) by a high-pressure (1,200 bar) homogenization technology. Subcutaneously injection of RBD-BBVs could stimulate SARS-CoV-2-specific immune responses in murine models. Irene et al. (2019) used lipoprotein transport pathways to prepare MVs with heterologously expressed proteins. The coding genes of five *S. aureus* antigens, namely, Hla_H__35__L_, SpA_KKAA_, LukE, Csa1A, and FhuD2, were fused with the lipoprotein leader sequence, and these recombinant proteins were expressed in *E. coli* BL21-Δ*ompA*Δ*msbB*Δ*pagP*. Immunization with MVs derived from the engineered bacteria could protect mice from infection caused by *S. aureus* strain Newman (Irene et al., 2019).

The proteins carried by the MVs from G^+^ bacteria, such as *S. pneumoniae*, *M. tuberculosis*, and *S. aureus*, are highly immunogenic, and they can induce effective immune responses in animal models (Olaya-Abril et al., 2014; Prados-Rosales et al., 2014a; Choi et al., 2015; Bitto and Kaparakis-Liaskos, 2017). However, studies on the loading of heterologous antigens in G^+^ MVs are few, probably due to the thickened cell wall that may hamper the MV’s release from G^+^ bacteria. We have used a 3 × FLAG protein as an exogenous antigen molecule to test the delivery potential of *S. aureus* proteins by fusing several protein genes with the coding sequence of 3 × FLAG (Yuan et al., 2018). In the *S. aureus* strain RN4220, at least four candidates, namely, PdhB, Eno, Mntc, and PdhA, can be fused with heterologous 3 × FLAG. The fusion proteins can be displayed on MVs observed with immunoelectron microscopy. Furthermore, when NS1 and two degenerated protective antigens EDIIIconA and EDIIIconB of dengue virus were individually fused to Mntc, Eno, and PdhB encoding genes in *S. aureus* strain RN4220-Δ*agr*, the resultant MVs could induce protective antibodies against all four serotypes of the dengue virus (Benz and Schmidt, 1989; Yuan et al., 2018). Chen G. et al. (2020) constructed multiple antigen vaccines by coating *S. aureus* MVs on the indocyanine green (ICG)-loaded magnetic mesoporous silica nanoparticles (MSN) to achieve EV/ICG/MSN, which could improve CD8^+^ T cell responses by activating MHC-I expression and promote CD4^+^ T cell response by up-regulating the expressions of costimulatory molecules, MHC-II molecules, and cytokines. Such engineered vaccines delivered by bacterial MVs could prevent skin/soft tissue infections caused by *S. aureus* and reduce bacterial invasion (Chen G. et al., 2020). The MV-enriched components are potential carrier molecules to load heterologous antigens to the MVs, however, the loading efficiency may be varied and must be experimentally determined during the development of a vaccine delivery vehicle.

### Anti-infective Drug Delivery

Synthetic nanomaterials, such as polymers, liposomes, and metal nanoparticles, have been extensively studied as drug carriers (Lin et al., 2018). However, the interaction between such delivery materials and the mammal cells is ambiguous. Bacterial MVs are made up of a bilayer lipid membrane and can effectively interact with living cells by passively accumulating at the site of infection or actively targeting host immune cells, such as macrophages (Schlatterer et al., 2018; Wang et al., 2020b). MVs can be loaded with therapeutic drugs and serve as engineered treatment agents during active infection. Gao et al. (2019) found that a nanoparticle coated with bacterial MVs (NP@EV) is an active targeting carrier that can successfully be delivered to the infectious sites *in vitro* and *in vivo*. The NP@EV carriers prepared with *S. aureus* MVs are internalized more efficiently by the *S. aureus*-infected macrophage than the un-infected counterpart. NP@EV particles constructed with *E. coli* MVs are more effectively internalized by the *E. coli*-challenged macrophage than the un-infected counterpart, but not the NP@EV agents prepared with *S. aureus* MVs (Gao et al., 2019). In mice with *S. aureus* infections, the intravenously injected rifampicin-loaded NP@EV particles constructed with *S. aureus* MVs conferred striking therapeutic efficiency (Gao et al., 2019). The active targeting abilities of bacterial MVs to their homologous pathogen-infected cells make them a promising drug delivery platform for engineering drug nanoparticles to control bacterial infections, especially infections caused by drug-resistant superbugs. However, owing to the intrinsic complexity, size heterogeneity, and component inhomogeneity, the inherent risks of bacterial MV as a drug-loaded platform are higher than those of well-established liposomes (Herrmann et al., 2021). Drug-loading methods for MVs should be also optimized and initiated in the industrial production.

### Anti-tumor Drug Delivery

The role of bacterial MVs in anti-tumor drug delivery for treatment has attracted attention in recent years (Cao and Liu, 2020). Compared with most traditional drug delivery vehicles, MVs have several unique advantages as anti-tumor drug carriers for cancer treatment. Firstly, bacterial MVs have a large anti-tumor drug loading space like synthetic nanoparticles. The protein drugs such as fibroblast growth factors were presented on the surface of MVs (Huang et al., 2020), while siRNA drugs were loaded into MV lumen by electroporation (Gujrati et al., 2014). Secondly, nano-sized MVs are more rigid and they present less leakage during host circulation than traditional liposomes. Thirdly, bacterial MVs have natural cell targeting capabilities. MVs derived from *E. coli* and *S. Typhimurium* contain adhesin molecules which could make MVs to be recognized and endocytosed by cells in the gastrointestinal tract (Benz and Schmidt, 1989; Liu et al., 2016a). Lastly, bacterial MVs carry various immune-stimulating molecules such as LPS that can initiate anti-tumor immune response (Cao and Liu, 2020). Chen Q. et al. (2020) coated MVs produced by *Salmonella* on drug-loaded polymeric micelles to activate the host’s immune response for cancer immunotherapy. The engineered MVs provided effective immune protection against melanoma and significantly inhibited the growth of tumors, thereby prolonging the survival of melanoma mice (Chen Q. et al., 2020).

Bacterial MVs have been widely used to deliver different kinds of anti-tumor drugs, including chemo-therapeutic agents, thermo-therapeutic molecules, and immuno-stimulatory elements (Liu et al., 2013; Chen Q. et al., 2020; Huang et al., 2020). A clinic trial has revealed that paclitaxel-loaded bacterial MVs were safe in patients carrying solid tumors and exhibited a modest clinical treatment efficacy (Solomon et al., 2015). The doxorubicin-carried MVs could deliver drugs to the neuroblastoma *in vivo* (Sagnella et al., 2018). Gujrati et al. (2019) genetically modified *E. coli* K12 to generate MVs loaded with biopolymer-melanin, and the resulting MVs were successfully used for optoacoustic imaging and thermal therapy of mice carrying subcutaneous 4T1 mammary gland tumors. Genetic modification technology was also applied to *E. coli* DH5α to assemble MVs surface with murine fibroblast growth factor (FGF). The persistent autoantibodies against FGF could be stimulated in mice after three subcutaneous injections of the engineered MVs, and the growth and metastasis of TC-1 and B16F10 xenograft tumors were effectively inhibited in mice vaccinated with MVs (Huang et al., 2020). Overall, these studies above demonstrate that bacterial MVs can provide targeted loading and delivery of a range of anti-tumor drugs in a highly effective way.

## Perspectives

The nano-sized and lipid membrane structure of bacterial MVs make them become a promising platform for broad application prospects. Genetic modifications of target bacteria have been verified to be one of the most effective strategies to optimize bacterial MVs for applications. Detoxification of bacterial MVs by consecutively deleting virulence factor genes one by one is inefficient, manipulation of pathogenicity island or global regulators that control the expression of virulence factors provides new options. Studies have shown the non-homogenous distribution of antigens and lipids in bacterial MVs. Further investigations to uncover the mechanisms of vesiculation would facilitate the generation of engineered MVs enriched in ideal components for application. Furthermore, the quantification of bacterial MVs is complicated and varies in different studies, including Braford protein assay, Lowry protein assay, phospholipid assay, KDO assay, FM1-43 assay, ^14^C-labeled radioactive assay, *etc*. (Table 2). A universal methodology to quantify bacterial MVs would be required for the fields of MV research and application. In addition, the biological safety, loading capacity, relative purity, structural homogeneity, cell-targeting ability, and tissue distribution of MV-coated particles need further investigation for creating more effective MV agents and improving human health.

## Author Contributions

RZ and XR contributed to the conception and design of the review. LQ wrote the first draft of the manuscript. YR edited the manuscript and the figures. XR, RZ, and KZ critically read and corrected the manuscript. All authors contributed to manuscript revision, editing, and approved the submitted version.

## Conflict of Interest

The authors declare that the research was conducted in the absence of any commercial or financial relationships that could be construed as a potential conflict of interest.

## Publisher’s Note

All claims expressed in this article are solely those of the authors and do not necessarily represent those of their affiliated organizations, or those of the publisher, the editors and the reviewers. Any product that may be evaluated in this article, or claim that may be made by its manufacturer, is not guaranteed or endorsed by the publisher.
